# Trifid nasopalatine canal: case report of a rare anatomical variation

**DOI:** 10.1016/j.radcr.2025.10.004

**Published:** 2025-11-08

**Authors:** Saba Khorram, Fereshteh Hayatimotlagh, Bita Heydarzadeh

**Affiliations:** Department of Oral and Maxillofacial Radiology, School of Dentistiry, Shahid Beheshti University of Medical Sciences, Tehran, Iran

**Keywords:** Nasopalatine canal, Cone-beam computed tomography, Anatomical variation, Dental implant

## Abstract

The nasopalatine canal (NPC) is a critical anatomical structure in the premaxilla, housing neurovascular components essential to maxillofacial surgery. While typically presenting as a single or bifurcated canal, rare variations such as a trifid configuration can complicate dental implant placement. This case report describes an incidental finding of a trifid NPC in a 38-year-old male during a pre-implant cone-beam computed tomography (CBCT) evaluation. The CBCT revealed 3 distinct canals with symmetrical distribution, visible in axial, coronal, and sagittal views, converging at the incisive foramen without associated pathologies. This rare anatomical variant necessitated adjustments in the surgical plan to minimize risks of nerve injury and ensure implant stability. The case underscores the importance of CBCT in identifying complex NPC morphologies for optimizing surgical outcomes and highlights the need for further research into the prevalence and clinical implications of such variations.

## Introduction

The premaxilla is a critical anatomical region in the maxillofacial complex, frequently subjected to trauma and tooth loss, which often necessitates surgical interventions such as dental implant placement [1,2]. A key anatomical landmark in this region is the nasopalatine canal (NPC), also referred to as the incisive canal or anterior palatine canal. Positioned in the midline of the maxilla posterior to the central incisors, the NPC serves as a conduit connecting the nasal cavity floor to the roof of the oral cavity [3,4]. At its inferior aspect, the canal opens into the oral cavity through the incisive foramen, which exhibits a funnel-shaped morphology beneath the incisive papilla. Superiorly, the canal is divided by the nasal septum into 2 foramina, known as the nasopalatine or Stenson’s foramina [[5], [6], [7]]. The NPC houses important structures, including the nasopalatine nerve, the terminal branch of the nasopalatine artery, fibrous connective tissue, adipose tissue, and minor salivary glands [8,9].

Advanced imaging modalities, such as cone-beam computed tomography (CBCT), have revolutionized the assessment of NPC anatomical variations, providing detailed 3-dimensional visualization that enhances diagnostic accuracy [1,3]. Understanding the morphology, number, and size of the NPC is of paramount importance in premaxillary surgeries, particularly for procedures such as local anesthesia administration in the anterior maxilla and dental implant placement, where anatomical variations can significantly impact surgical outcomes [10]. While the NPC typically presents as a single or bifurcated canal, rare variations, including additional canals, have been documented in a limited number of studies [8]. Among these, the trifid NPC represents an exceptionally uncommon anatomical variant, with significant implications for surgical planning due to its potential to complicate nerve preservation and implant stability [8,10]. This case report describes a rare instance of a trifid nasopalatine canal identified through CBCT imaging during a pre-implant evaluation, highlighting its clinical relevance in the context of dental implant surgery.

### Case report

A 38-year-old male patient was referred to the oral and maxillofacial radiology department for a pre-implant evaluation of the left anterior maxilla. The patient had an unremarkable medical history with no systemic conditions or symptoms, including pain, swelling, or sensory disturbances in the maxillofacial region. Intraoral examination revealed normal soft tissues, dentition, and palate, with no abnormalities noted in the incisive papilla, such as inflammation or deviation.

**Fig. 1:**
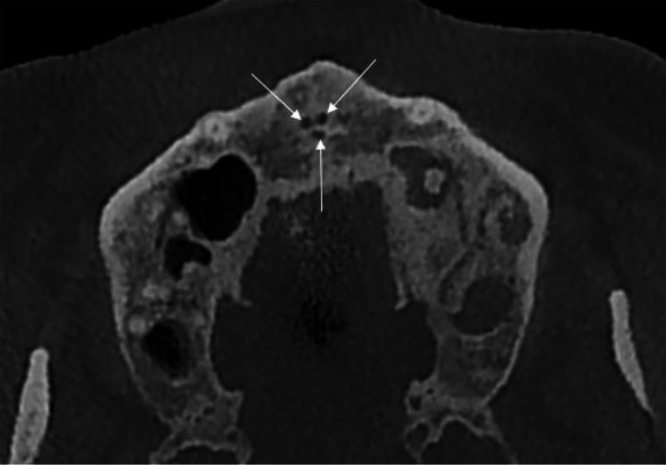
Axial CBCT image illustrating the symmetrical distribution of all 3 canals of the trifid nasopalatine canal (arrows) and their relationship with the surrounding maxillary structures.

A CBCT scan was ordered to assess the premaxillary region prior to dental implant placement in the left maxilla, as accurate evaluation of anatomical landmarks like the NPC is essential for surgical planning [10]. During the radiological assessment, an incidental finding of a rare anatomical variation was identified: a trifid nasopalatine canal. The CBCT images provided a comprehensive 3-dimensional evaluation of the NPC, which is crucial for identifying morphological variations [1,3]. The axial view (Fig. 1) clearly demonstrated the trifid configuration, revealing all 3 canals with a symmetrical distribution and their relationship with adjacent structures, including the maxillary incisors, which showed no signs of root resorption or abnormal proximity to the canals [9].

**Fig. 2:**
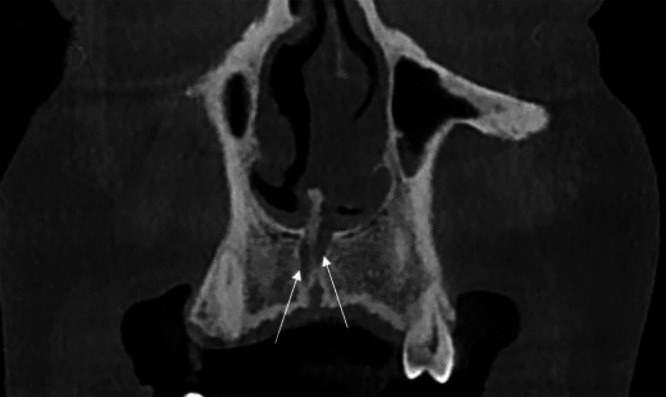
Coronal CBCT image of a 38-year-old male patient revealing a trifid nasopalatine canal, identified incidentally during pre-implant assessment. The image shows 2 of the 3 channels (arrows) separated by a bony septum, originating from the nasal floor and converging toward the incisive foramen, with the right and posterior canals aligned in this section.

In the coronal view (Fig. 2), the NPC presented as 2 distinct channels, separated by a thin bony septum, originating from the nasal floor and converging toward the incisive foramen. Due to the non-linear alignment of the canals, only 2 of the 3 canals were visible in this plane, with the right and posterior canals aligned in this particular section.

**Fig. 3:**
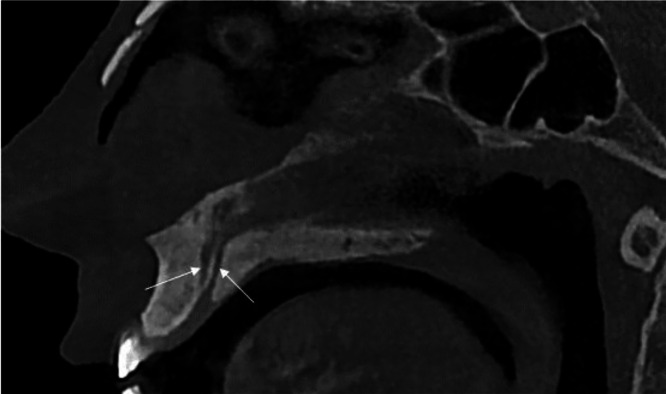
Sagittal CBCT image depicting the vertical course of the trifid nasopalatine canal, extending from the nasal floor to the incisive foramen with a funnel-shaped morphology at the oral opening. In this plane, the left and posterior canals are aligned and visible (arrows).

The sagittal view (Fig. 3) illustrated the vertical trajectory of 2 canals, extending from the nasal floor to the incisive foramen, with a characteristic funnel-shaped morphology at the oral opening, as described in anatomical studies [5]. In this plane, the left and posterior canals were aligned and visible.

No associated pathologies, such as nasopalatine duct cysts, bone erosion, or soft tissue abnormalities, were observed in the surrounding bone or adjacent structures, aligning with findings from prior studies on NPC variations [2,6]. The trifid NPC is an exceptionally rare anatomical variant, with only a few cases reported in the literature, underscoring its clinical relevance in surgical contexts [8]. Although the planned implant placement was in the left maxilla, the midline trifid NPC could still pose indirect risks, such as nerve injury, due to potential variations in nerve distribution or anatomical extensions affecting the surgical site [10]. Furthermore, the proximity of the canals to the planned implant site necessitates careful consideration to prevent encroachment during surgery [2].

Given the incidental and asymptomatic nature of the finding, no immediate intervention was required for the NPC itself. However, the trifid NPC was identified as a critical anatomical consideration for the planned implant placement in the left maxilla. The patient was informed of the finding, and a modified surgical approach was recommended, potentially involving adjustments in implant angulation or position to ensure a safe distance from the NPC and minimize complications [2]. Periodic follow-up was advised to monitor the region for any future changes.

## Discussion

The premaxilla is a pivotal region for maxillofacial surgeries, such as dental implant placement, due to its aesthetic and functional significance [9]. The nasopalatine canal, also known as the incisive canal, is a critical anatomical landmark in this region, located in the midline posterior to the central incisors [2]. Its neurovascular contents, including the nasopalatine nerve and artery, make it a structure of concern during surgical interventions [1].

The incidental finding of a trifid NPC in a 38-year-old male patient during a pre-implant CBCT evaluation highlights the importance of advanced imaging in detecting anatomical variations. CBCT provides high-resolution, 3-dimensional visualization, enabling clinicians to identify complex NPC morphologies that could complicate procedures like implant placement or orthognathic surgery, thus reducing risks such as nerve injury or implant failure [11].

The normal anatomy of the NPC typically features a single or bifurcated canal, extending from the nasal floor to the incisive foramen, with a funnel-shaped oral opening beneath the incisive papilla [2]. It houses the nasopalatine nerve, terminal branches of the nasopalatine artery, connective tissue, adipose tissue, and minor salivary glands [12]. Recent studies report an average NPC length of 10-12 mm and a diameter of 3-5 mm at the incisive foramen, with variations influenced by age, gender, and dental status [4].

In this case, the trifid NPC, characterized by 3 distinct channels converging at the incisive foramen, represents a significant deviation from the norm, as observed in the axial, coronal, and sagittal CBCT views [8]. Recent literature has documented rare NPC variations, providing a basis for comparison with our findings.

Torres et al. reported a trifid NPC, noting its potential to increase the risk of neurovascular injury during implant placement due to multiple canal branches [8]. Safi et al. evaluated morphologic variations of the NPC using CBCT, highlighting the presence of rare configurations that may complicate surgical planning [1]. Etoz et al. described variations in NPC morphology, emphasizing the need for detailed preoperative imaging to identify such anomalies [2].

Our case aligns with these reports in terms of the rarity of the trifid configuration but is unique in its asymptomatic presentation and absence of associated pathologies, such as nasopalatine duct cysts, which were not observed in the CBCT images [6]. The symmetrical distribution of the 3 canals in our axial CBCT view contrasts with the complex morphologies reported by Safi et al., where some canal variations were less clearly visualized in certain planes [11]. These comparisons underscore the critical role of CBCT in identifying subtle anatomical variations that may be missed by conventional imaging [13].

This study has several limitations. As a single case report, it cannot estimate the prevalence of trifid NPCs across populations or ethnic groups. The retrospective nature of the CBCT analysis restricted our ability to evaluate long-term clinical outcomes or functional implications of the trifid NPC [10]. Additionally, the non-linear alignment of the canals, which limited visibility of 1 canal in certain CBCT planes, suggests that standard CBCT protocols may not fully capture complex morphologies [14].

Future studies should involve larger, multicenter cohorts to determine the prevalence of trifid and other rare NPC variations, particularly across diverse populations [13]. Advanced imaging techniques, such as microCT or enhanced 3-dimensional reconstruction, could improve visualization of intricate canal structures, as demonstrated in ex vivo studies [7]. Embryological and genetic studies are also recommended to investigate the developmental origins of trifid NPCs, potentially linked to aberrant nasopalatine duct fusion during fetal development [5]. Prospective studies assessing surgical outcomes in patients with trifid NPCs would further inform clinical management strategies [10].

In conclusion, the trifid nasopalatine canal is a rare anatomical variation with significant implications for maxillofacial surgery. This case emphasizes the indispensable role of CBCT in preoperative planning to detect such anomalies and optimize surgical outcomes. Clinicians should routinely employ advanced imaging to mitigate risks associated with NPC variations, and future research should focus on elucidating the prevalence, etiology, and clinical impact of these rare morphologies.

## Patient consent

The patient provided informed consent for his clinical information and images to be included in this case report. He understood the purpose of the publication, how his privacy would be protected, and agreed voluntarily to share his data for research purposes.
